# Detection of potentially pathogenic bacteria on cell phones of hospital and university-based populations in Curitiba, southern Brazil. A cross-sectional study

**DOI:** 10.1590/1516-3180.2018.044305072019

**Published:** 2019-09-23

**Authors:** Andressa Siqueira Jansen, Giuliano Carlo Balbinot, Alessandra Vale Daur, Andrei Christian Ferri da Silva, Keite Silva Nogueira, Thaiz Fernandes, Camila Marconi

**Affiliations:** I Undergraduate Student, Biomedical Sciences Course, Universidade Federal do Paraná (UFPR), Curitiba (PR), Brazil.; II Undergraduate Student, Biomedical Sciences Course, Universidade Federal do Paraná (UFPR), Curitiba (PR), Brazil.; III MSc. Technical Manager, Laborclin Produtos para Laboratórios, Pinhais (PR), Brazil.; IV MSc. Technical Manager, Laborclin Produtos para Laboratórios, Pinhais, PR, Brazil.; V PhD. Adjunct Professor, Department of Basic Pathology, Universidade Federal do Paraná (UFPR), Curitiba (PR), Brazil.; VI BSc. Master’s Student, Postgraduate Program, Department of Basic Pathology, Universidade Federal do Paraná (UFPR), Curitiba (PR), Brazil.; VII PhD. Adjunct Professor, Department of Basic Pathology, Universidade Federal do Paraná (UFPR), Curitiba (PR), Brazil.

**Keywords:** Cell phone, Methicillin-resistant *Staphylococcus aureus*, Enterobacteriaceae

## Abstract

**BACKGROUND:**

Cell phones have become indispensable for professional activities, including healthcare. Thus, they are possible sources of bacterial contamination. There is a scarcity of data in the literature regarding identification of risk factors for contamination of cell phones with pathogenic bacteria.

**OBJECTIVE:**

To compare the prevalence rates of *Staphylococcus aureus (S. aureus)*, methicillin-resistant *S. aureus* (MRSA) and/or Enterobacteriaceae on cell phones belonging to hospital healthcare staff and university students in Curitiba, Paraná, Brazil, and to identify variables associated with such contamination.

**DESIGN AND SETTING:**

Cross-sectional study conducted in a public university’s referral hospital and lecture buildings in 2017.

**METHODS:**

We sampled the surface of cell phones using the dipslide method, with Baird-Parker agar and *Escherichia coli*-coliform chromogenic (ECC) agar. We assessed the population’s sociodemographic, behavioral and hygiene characteristics through interviews. Possible presence of *S. aureus* colonies was confirmed using agglutination tests, with evaluation of methicillin sensitivity. Colonies in ECC medium were counted. Stepwise logistic regression (forward P < 0.15) was performed to identify characteristics associated with bacterial contamination.

**RESULTS:**

The prevalence rates of *S. aureus*, MRSA and Enterobacteriaceae were, respectively, 32%, 4% and 3%. No difference was found between the hospital and university-based populations (P > 0.05). The only variable associated with bacterial contamination was the use of cloth/velvet/leather phone cases (odds ratio: 2.92; 95% confidence interval: 1.08-7.91).

**CONCLUSIONS:**

Potentially pathogenic bacteria were prevalent on the cell phones of this hospital and university population. Use of phone cases made of cloth-like material should be discouraged, especially in hospital settings.

## INTRODUCTION

The number of cell phones users is estimated to be five billion, which corresponds to more than two thirds of the world’s population.^1^ Internet access is one of the factors responsible for the increasing number of users.^2^ Over time, these devices have become indispensable not only for personal but also for professional life, since they allow efficient and quick communication, along with online searches.^3^^,^^4^^,^^5^

In hospitals, cell phones have also been widely used for sharing clinical information and the results from laboratory tests, diagnostic imaging and so on. In addition, several applications (“apps”) with clinical utility have been developed for, but not limited to, drug dosage calculations, request codes for laboratory tests and access to scientific publications.^3^^,^^5^^,^^6^ Thus, it is now impossible to dissociate the use of cell phones from healthcare assistance, especially in hospital settings. However, over the last decade, it has been pointed out in the literature that they might be considered to be a source of bacterial contamination, both in hospital and in community settings.^7^^,^^8^

Studies have shown that the skin colonizer *Staphylococcus aureus (S. aureus)* is the most frequent pathogenic bacterial species isolated from cell phones. Additionally, they have shown that methicillin-resistant *Staphylococcus aureus* (MRSA) may be found among the isolates.^4^^,^^5^ The pathogenic potential of MRSA is unquestionable, especially with regard to nosocomial infections. Moreover, although cell phone contamination caused by members of the family Enterobacteriaceae is less frequent than contamination due to MRSA, it is not rare. Species such as *Klebsiella pneumoniae* and *Escherichia coli* have been reported as contaminants of these devices and are undoubtedly important infectious agents.^4^^,^^9^^,^^10^

Although studies aiming to detect bacterial contamination of cell phones have already been conducted in several countries, they mostly focused on microbiological findings. Thus, those studies did not provide any information regarding the characteristics of the population that were associated with detection of pathogenic bacteria on these devices.^5^^,^^10^^,^^11^^,^^12^ Additionally, to the best of our knowledge, there are no studies in the scientific literature showing information regarding cell phone contamination in Brazilian hospitals.

## OBJECTIVE

Our aim was to compare the prevalence and loads of *S. aureus* (including MRSA) and Enterobacteriaceae on the cell phones belonging to healthcare professionals at a referral hospital with those on phones belonging to university students, in Curitiba, Paraná, Brazil. Additionally, we aimed to test associations between population characteristics and such contamination.

## METHODS

### Study design, ethics and sampling

This study was approved by the Ethics Committee of the Federal University of Paraná (Universidade Federal do Paraná, UFPR), under the number 1.858.500, on December 9, 2016. From January to September 2017, we cross-sectionally screened the cell phones of 300 participants, who were recruited in equal numbers (n = 150) in two enrollment settings.

One of the enrollment centers was the Hospital das Clínicas (Clinics Hospital, HC) of UFPR, which is a referral hospital in Curitiba, state of Paraná, Brazil. At HC-UFPR we enrolled multidisciplinary healthcare professionals who were attending the postgraduate course “Multiprofessional Integrated Residency Program of the HC/UFPR”. This is a two-year specialization course that is taken by physicians, nurses, physiotherapists, dentists and psychologists, among others, who have close contact with hospital patients during their activities for obtaining their specialization degree. The participants were approached by the research team during their coffee or lunch break.

The second enrollment center was the Biological Sciences Sector (BSS) of UFPR, in Curitiba, Brazil. University students were approached in classroom halls while waiting for their next lecture. They were taking undergraduate health-related courses (medicine, biomedical sciences, physiotherapy, nursing and dentistry) at BSS-UFPR. Some of the students may also have been attending HC-UFPR for lectures and/or extracurricular activities. These participants did not see patients by themselves and were not performing any type of procedure at inpatient or outpatient clinics, since they were only accompanying local medical staff as part of their extracurricular activities. Among the undergraduates of the medicine course, none of them had started their internship at the time of enrollment.

### Approaches to participants and sample collection

Three members of the research team (ASJ, GCB and TF) visited each enrollment center once a week and included similar numbers of participants per visit. When approaching potential participants, we explained the objectives of the study. Upon giving agreement to participate, these individuals signed a consent statement. None of the participants approached refused to participate. Before any sampling procedure was conducted, the participants individually answered a structured questionnaire. The questions sought information about their habits regarding the places where they used the phone (including the bathroom and bedroom), frequency of cleansing their hands and phone and the cleansing products used, among other information.

Only the participants themselves held their devices during the sampling procedure, in order to avoid contamination with the researchers’ skin microbiota. Samples were obtained by allowing contact between the whole surface of the device and the two sides of the commercial dipslide Nutrilab P (Laborclin, Pinhais, Paraná, Brazil), which was coated with Baird-Parker agar and *Escherichia coli*-coliform chromogenic agar (ECC) on each face.

### Sample analysis

The samples were transported to the laboratory within two hours after collection and were immediately incubated at 37 °C for 48 hours. In the presence of any growth, colonies on both faces of Nutrilab P were counted and recorded according to their morphology. The numbers of similar colonies retrieved in each medium were divided by the dipslide medium area (= 8.5 cm^2^) to obtain the number of colony-forming units (CFU) per cm^2^.

We considered that areas of black or gray color surrounded by a lipase halo on Baird-Parker agar were potentially colonies of *S. aureus*. To make a positive identification of *S. aureus*, these strains were tested for catalase production and confirmed using the latex agglutination-based test StaphclinLatex (Laborclin, Pinhais, Paraná, Brazil), in accordance with the manufacturer’s instructions.

We further tested *S. aureus* isolates for cefoxitin susceptibility by means of disk diffusion, as standardized by the Clinical and Laboratory Standards Institute (CLSI), 2015.^13^ When the cefoxitin inhibition zone diameter was < 22 mm, the strains were identified as MRSA.^14^ We also identified all colony types counted on ECC agar at species level using the phenotypic tests provided through the enteroBacterias kit (Laborclin, Pinhais, Paraná, Brazil), in accordance with the manufacturer’s instructions.

### Statistical analysis

In the data analyses, variables regarding cell phone use and hygiene habits were compared between participants from the two enrollment settings using the Mann-Whitney nonparametric test and chi-square test for, respectively, continuous and categorical variables. The number of positive cultures and the number of colonies grown were compared using, respectively, the chi-square and Mann-Whitney tests, also according to the enrollment setting.

Additionally, univariate logistic regression models were constructed to assess any associations between the variables and the presence of any cell phone contamination (by *S. aureus* and/or Enterobacteriaceae). Crude and enrollment setting-adjusted odds ratios (OR) were estimated, along with their corresponding 95% confidence intervals (CI). Lastly, multivariable logistic regression analysis was carried out using a forward stepwise model selection process (variables retained at P-values ≤ 0.15), to identify variables independently associated with contamination.

All the statistical analyses were performed using Stata (Statacorp LLC, College Station, TX), considering P-values < 0.05 to be significant.

## RESULTS

The median age of the 300 participants was 23 years, and most of them were female (n = 236; 78.7%) (Table 1). All the information on the participants’ behavioral characteristics and hygiene habits that was acquired through interviews is shown in Table 1.

**Table 1.** Sociodemographic data, behavioral characteristics and hygiene habits of the study participants: overall and according to enrolment setting

	Overall (n = 300)	BSS-UFPR (n = 150)	HC-UFPR (n = 150)	P-value
Age (years), median (min-max)	23 (17-74)	20 (17-39)	27.5 (21-74)	< 0.0001
Gender				
Male	64 (21.3%)	41 (27.3%)	23 (15.3%)	0.01
Female	236 (78.7%)	109 (72.7%)	127 (84.7%)	
Places in which cell phone was used				
In all rooms of the house	283 (94.3%)	141 (94.0%)	142 (94.7%)	0.80
In bed	283 (94.3%)	146 (97.3%)	137 (91.3%)	0.03
Water and soap available in all bathrooms used	102 (34.0%)	25 (16.7%)	77 (51.3%)	< 0.0001
Regular use of hand sanitizer (gel with alcohol)	209 (69.7%)	75 (50.0%)	133 (88.7%)	< 0.0001
Daily use of facial cream/sunscreen/foundation	210 (70.0%)	95 (63.3%)	115 (76.7%)	0.01
Day(s) at hospital facilities, per week				
None	83 (27.7%)	82 (54.7%)	1 (0.7%)	< 0.0001
1 day	44 (14.7%)	42 (28.0%)	2 (1.3%)	
2 or more days	173 (57.7%)	26 (17.3%)	147 (98.0%)	
Hospital settings regularly entered^a^				
None	113 (37.3%)	113 (75.3%)	0 (0.0%)	--
Outpatient clinics	107 (35.7%)	20 (13.3%)	87 (58.0%)	< 0.0001
Inpatient clinics	130 (43.3%)	18 (12.0%)	112 (74.7%)	< 0.0001
Operation rooms	47 (15.7%)	0 (0.0%)	47 (31.3%)	--
Intensive care units	25 (8.3%)	0 (0.0%)	25 (16.7%)	--
Other ^b^	56 (18.7%)	12 (8.0%)	44 (29.3%)	< 0.0001
Frequency of cell phone cleansing				
Never	164 (54.7%)	105 (70.0%)	59 (39.3%)	< 0.0001^c^
At least once	136 (45.4%)	45 (30.0%)	91 (60.7%)	
Daily	26 (8.7%)	5 (3.3%)	21 (14.0%)	
Weekly	65 (21.7%)	21 (14.0%)	44 (29.3%)	
Monthly	45 (15.0%)	19 (12.7%)	26 (17.3%)	
Products used for cell phone cleansing				
None	164 (54.7%)	105 (70.0%)	59 (39.3%)	< 0.0001
Yes, using alcohol (gel or a 70% [v/v] solution)	104 (34.7%)	33 (22.0%)	71 (47.7%)	
Yes, using other products (soap, wipes, cloth or paper)	32 (10.6%)	12 (8.0%)	20 (13.3%)	
Material of the phone case				
None	74(24.7%)	38 (25.3%)	36 (24.0%)	0.46
Plastic/silicone/rubber	209 (69.7%)	106 (70.7%)	103 (68.7%)	
Cloth/velvet/leather	17 (5.7%)	6 (4.0%)	11 (7.3%)	

BSS = Biological Sciences Sector; UFPR = Universidade Federal do Paraná; min-max = minimum-maximum; HC = Hospital of Clinics; -- Not calculated; ^a^Sum may be greater than 100%, because the participants mostly entered more than one hospital setting; bLaboratories, administrative offices, pharmacy and others; cComparison between “never” and “at least once” categories.

Nearly all the participants (94.3%) reported that they used their cell phones in all rooms of the house, including in bed. Furthermore, the data stratified according to enrollment setting in Table 1 shows that a significantly higher proportion of the participants enrolled at the university hospital (HC-UFPR) reported having access to bathrooms equipped with water and soap for hand washing at all times (51.3%) and making regular use of hand sanitizer (gel with alcohol) (88.7%).

Regarding cell phone cleansing habits, 70% of the students at BSS reported that they had never cleaned their device. This proportion was significantly lower among the participants enrolled at the hospital (39.3%). The most common product used for phone cleansing was alcohol solution (gels or liquid). At both enrollment sites, nearly 70% of the participants used phone cases made of smooth materials such as plastic, silicone or rubber, while approximately 5% used a cloth-like case (including cloth, velvet or leather materials).

The prevalence rates of contamination according to the enrolment setting are displayed in Table 2. The overall positivity for *S. aureus* was 32% and did not differ between enrollment sites. Among the 46 cases of *S. aureus* isolated from the students’ phones, 5 (3.3%) were methicillin-resistant. The proportion of MRSA among the isolates from the hospital population was higher (n = 8; 5.3%) but did not reach statistical significance. The positivity rate for Enterobacteriaceae in the hospital population was twice the rate among the students, but did not differ statistically (Table 2). Regarding the comparison between the numbers of colonies yielded from the two study groups, no difference was observed in relation to any of the microorganisms.

**Table 2.** Comparison of frequency and number of CFU of *Staphylococcus aureus* and Enterobacteriaceae isolated from participants’ cell phones, between enrollment sites

	BSS/UFPR (n = 150)	HC/UFPR (n = 150)	P-value
*Staphylococcus aureus*			
Positivity n (%)	46 (30.7%)	51 (34.0%)	0.54^a^
CFU/cm^2^, median (min-max)	0.6 (0.1-8.8)	0.7 (0.1-14.1)	0.95^b^
MRSA			
Positivity n (%)	5 (3.3%)	8 (5.3%)	0.57^c^
CFU/cm^2^, median (min-max)	0.6 (0.2-2.4)	0.9 (0.1-2.0)	0.83^b^
Enterobacteriaceae			
Positivity n (%)	3 (2.0%)	6 (4.0%)	0.50^c^
CFU/cm^2^, median (min-max)	0.5 (0.1-0.5)	0.1 (0.1-0.4)	0.22^b^
*Staphylococcus aureus* and/or Enterobacteriaceae			
Positivity n (%)	49 (32.7%)	54 (36.0%)	0.54^a^
CFU/cm^2^, median (min-max)	0.6 (0.1-8.8)	0.6 (0.1-14.1)	0.74^b^

CFU = colony-forming units; UFRP = Universidade Federal do Paraná; min-max = minimum-maximum; SBS = Biological Sciences Sector; HC = Clinics Hospital; MRSA = methicillin-resistant *Staphylococcus aureus* (resistant if zone diameter < 22 mm in cefoxitin disk test); v/v = volume/volume.

^a^Chi-square test; ^b^Mann-Whitney test; ^c^Fisher exact test.

Table 3 shows the results from association tests between positive cultures for *S. aureus* and/or Enterobacteriaceae and the characteristics assessed in the study population. Three different association analyses were performed: crude, adjusted for enrollment setting and multivariable. All of them showed very similar results. None of the variables tested were associated with colonization with *S. aureus* and/or Enterobacteriaceae except for the use of phone cases made of cloth-like material (cloth, velvet or leather).

**Table 3.** Odds ratio and 95% confidence interval for the association of positivity in cultures for *Staphylococcus aureus* and/or Enterobacteriaceae with sociodemographic, behavioral and hygiene habits

	Crude	Enrollment setting-adjusted	Multivariable
Age	1.00 (0.99-1.03)	1.01 (0.98-1.03)	--
Gender			
Male	1.00	1.00	--
Female	0.63 (0.34-1.17)	0.64 (0.34-1.19)	
Sees patients on regular basis^a^			
No	1.00	1.00	--
Yes	1.15 (0.71-1.86)	1.09 (0.59-2.00)	
Cell phone use in all rooms at home			
No	1.00	1.00	--
Yes	0.57 (0.21-1.52)	0.56 (0.21-1.51)	
Water and soap available in all bathrooms used at work/study facilities			
No	1.00	1.00	--
Yes	1.38 (0.84-2.27)	1.36 (0.79-2.32)	
Regular use of hand sanitizer (gel with alcohol)			
No	1.00	1.00	--
Yes	1.11 (0.66-1.88)	1.05 (0.59-1.87)	
Day(s) at hospital facilities, per week^b^			
None or 1 day	1.00	1.00	--
2 or more days	1.10 (0.70-1.79)	0.92 (0.40-2.16)	
Daily phone cleansing			
No	1.00	1.00	--
Yes	0.68 (0.28-1.68)	0.64 (0.25-1.60)	
Phone cleansing with alcohol			
No	1.00	1.00	--
Yes	1.02 (0.62-1.68)	0.98 (0.58-1.64)	
Cloth/velvet/leather phone case			
No	1.00	1.00	1.00
Yes	2.92 (1.08-7.91)	2.87 (1.06-7.80)	2.92 (1.08-7.91)
Daily use of facial cream/sunscreen/foundation			
No	1.00	1.00	--
Yes	1.23 (0.72-2.09)	1.21 (0.71-2.06)	

-- variables not retained in the multivariable analysis (P-value > 0.10); ^a^intensive care units and in and outpatient clinics; ^b^at least for 2 hours excluding lecture rooms.

## DISCUSSION

Despite the constant use of cell phones in many daily activities of healthcare personnel, there is still no consensus regarding the best approach for cleansing frequency, products or techniques. Nonetheless, studies have consistently shown that cell phones can be a source of contamination in hospital environments and that decontamination practices conducted on these devices to decrease their bacterial load may also reduce the cross-contamination risk.^7^^,^^8^ Our data showed that the contamination rates were very similar to the prevalence of *S. aureus* carriage in individuals’ oral and nasal mucosae. We could have hypothesized that healthcare professionals may present even higher prevalence but, on the other hand, they were seen to be more inclined to take decontamination measures in relation to their cell phones and no such difference was noted.^13^

Another factor that could have contributed towards the similar contamination rates observed in the two groups was that most of the participants enrolled at HC/UFPR reported that they made regular use of hand sanitizers. The findings from a previous study corroborate this idea, since that study showed that the microbiota of the hands is the main source of contamination of cell phones.^6^ Hand sanitizer was also the most common product used for cell phone cleansing among the participants (104 out of 136; 76.4%) (data not shown). The efficacy of this product for reducing the microbial load on these devices has been acknowledged and has been recommended.^14^

Despite the notable rate of bacterial contamination of cell phones among the university students in our study (33%), this rate was lower than what has been reported in the literature. A study by Tagoe et al. found 100% prevalence of bacterial contamination on the cell phones of students in Ghana, while Zakai et al. showed 96% prevalence on the devices of students in Saudi Arabia.^8^^,^^15^ The contamination rate among healthcare professionals in our study (36%) was also lower than what has been reported in the literature, which has ranged from 74% to 91% in similar populations.^10^^,^^16^ The main reason for these discrepancies between our data and the reports in the literature is that we used two selective culture media, while the other studies were based on culturing in nutritionally enriched media, such as brain heart infusion, blood sheep agar and others.

Studies in the literature have mostly reported that the prevalence of bacterial contamination on students’ cell phones is higher than the prevalence on the devices of healthcare professionals.^8^^,^^10^^,^^14^^,^^17^ In part, this could be due to the more frequent decontamination procedures performed by hospital workers. However, those studies were performed either among students or among hospital staff and did not make comparisons between the two populations. Akinyemi et al. did compare the two populations and showed that the contamination rate of students’ cell phones was twice that of the devices belonging to healthcare workers.^7^ On the other hand, a study by Pal et al. agreed with our contamination rate by showing that it was greater among hospital staff than among students.^6^ It is worth mentioning that in our study, although 37 students (24.7%) did enter the hospital environment on a regular basis, they did not see patients by themselves because they were only undertaking extracurricular activities.

As expected, among the pathogenic bacterial species assessed in this study, the most prevalent of them was *S. aureus*. This result is similar to findings reported in the literature: a study by Rana et al. showed that *S. aureus* was the most prevalent species, not only on the cell phones of healthcare professionals, but also on those of the non-healthcare professionals.^4^ The positivity rate for MRSA found in our study was lower than what was previously reported on the cell phones of inpatients and both healthcare and non-healthcare professionals in Egypt and India.^4^^,^^5^ Regarding the species belonging to the Enterobacteriaceae family that were also assessed in our study, the positivity rates of 2% and 4% on the devices of students and healthcare professionals, respectively, were similar to those found in the literature. Studies by Pal et al. and Heyba et al. found, respectively, positivity rates of 6% and 7% on the cell phones of hospital staff members.^6^^,^^10^ One of the reasons for the lower prevalence of Gram-negative bacteria on cell phones may be their low tolerance towards desiccation and consequent reduced viability on the surface of cell phones.

The majority of our population (75%) used protective phone cases on their devices. According to a study by Tiwari et al., cell phones with protective cases show higher contamination rates than do those without cases, but they did not specify the types of materials used in the cases.^14^

Therefore, we now add to the literature the information that use of protective cases made of cloth-like material is independently associated with contamination of cell phones with potentially pathogenic bacteria. Hence, we suggest that the use of such covers should be discouraged, especially among healthcare staff and, even more importantly, those working in intensive care units or other clinics with especially critical patients. We also propose that this information should be addressed through guidelines regarding healthcare settings.

## CONCLUSION

The prevalence rates of MRSA and Enterobacteriaceae contamination on cell phones were found to be similar in community and hospital-based populations. The use of phone cases made of cloth, velvet or leather was independently associated with contamination. Therefore, the use of this type of case should be discouraged, especially in healthcare settings. Further studies are needed, including assessment of a wider range of variables with greater sample sizes. Such studies will better contribute towards knowledge of the behavioral or hygiene characteristics of populations that might increase the risk of contamination. Such knowledge would make it possible to develop important prophylactic strategies and ensure safe use of cell phones.
